# The Role of Tumor-Infiltrating B Lymphocytes in Colorectal Cancer Patients: A Systematic Review of Immune Landscape Evolution

**DOI:** 10.3390/cancers17182996

**Published:** 2025-09-13

**Authors:** Giorgiana Fagarasan, Vlad Fagarasan, Vasile Virgil Bintintan, George Calin Dindelegan

**Affiliations:** 1 Department of Anatomy and Embriology, “Iuliu Hatieganu” University of Medicine and Pharmacy, Str. Clinicilor 3-5, 400006 Cluj Napoca, Romania; amarinei.giorgiana@elearn.umfcluj.ro; 2 Department of Surgery, “Iuliu Hatieganu” University of Medicine and Pharmacy, Str. Clinicilor 3-5, 400006 Cluj Napoca, Romania; vasile.bintintan@umfcluj.ro (V.V.B.); george.dindelegan@umfcluj.ro (G.C.D.)

**Keywords:** tumor-infiltrating B lymphocytes, tumor microenvironment, b cell subtypes, tertiary lymphoid structures, prognostic impact, colon cancer, rectal cancer

## Abstract

Background: Previous studies have found that the presence and density of tumor-infiltrating T lymphocytes has significant prognostic value in colon and rectal cancer. However, there is a paucity of systematic reviews focusing on the role of tumor-infiltrating B lymphocytes (TIBLs) in colorectal cancer. The aim of this review was to assess the role of all known subsets of TIBLs implicated in colorectal cancer, as well as to identify the methodologies used for the identification of TIBLs and establish their prognostic value in the colorectal tumor microenvironment (TME). Methods: A systematic literature search was conducted from 2000 to 2025 using the PubMed (Medline), Embase and Cochrane Library databases. Preferred Reporting Items for Systematic Reviews and Meta-analysis (PRISMA) guidelines were followed to identify eligible studies. Results: A total of 32 original papers involving 5766 individual patients met the inclusion criteria. Several B cell types in the immune TME of colon and rectal cancer were identified, each with specific roles and prognostic value. Several distinct methodologies were used for the identification of these cells, according to specific detection protocols used by various institutions. Certain immunological pathways through which these cells may be involved in the carcinogenesis of colorectal tumors have also been identified, which may potentially lead to the development of novel immunotherapy targets. Conclusions: We concluded that TIBLs play a significant role in the immune microenvironment of colorectal cancer, providing theoretical evidence for potential biomarkers which may constitute a valuable target for individualized treatment.

## Simple Summary

This systematic review explores the role of tumor-infiltrating B lymphocytes (TIBLs) in the tumor microenvironment (TME) of colorectal cancer, addressing a gap in the immuno-oncology literature. A comprehensive literature search spanning 2000 to 2025 identified 32 eligible studies involving 5766 patients, in accordance with PRISMA guidelines. The findings revealed multiple B cell subsets, including plasma cells, memory B cells, and regulatory B cells. These subsets exhibited distinct roles and prognostic implications in the progression of colon and rectal cancer. The review also highlighted methodological variability in TIBL identification across studies. Furthermore, it identified immunological pathways through which TIBLs may influence carcinogenesis, suggesting their potential as novel immunotherapy targets. Overall, the review emphasizes the prognostic and therapeutic relevance of TIBLs, supporting their future use as biomarkers for personalized treatment strategies in colorectal cancer.

## 1. Introduction

### 1.1. Background

Colorectal cancer is the second most frequent cause of cancer-related death worldwide and one of the most commonly diagnosed malignant tumors in both sexes. Despite recent advances in the treatment of this disease and the decline in mortality due to early detection and management, there is an urgent need to identify more specific novel predictive and prognostic biomarkers in order to improve patient outcomes, as well as develop additional targeted therapies [1]. Immuno-oncology has recently emerged as a potential field of research, encompassing the idea that malignant tumors are recognized by the host immune system due to specific antigens, and their development can be modulated or stopped through a process known as immunosurveillance [2]. It is now widely accepted that the suppression of tumor development can be achieved through what is known as tumor microenvironment (TME) activity. The main components of the tumor microenvironment are the extracellular matrix and the cellular compartment, which includes fibroblasts, endothelial cells, and immune cells [3]. Previous studies have demonstrated that the presence and density of various tumor-infiltrating lymphocytes subtypes found in the tumor microenvironment have a significant influence on disease free survival, cancer specific survival and overall survival, demonstrating prognostic value as biomarkers for colorectal cancer, as well as other types of solid malignant tumors [4,5,6,7,8,9,10]. The identification of the tumor microenvironment has contributed significantly to the development of cancer immunotherapy as a major advancement in oncological treatment [11]. Due to this new emerging field, particular attention has been focused on the successful clinical application of chimeric antigen receptor T (CAR-T) cell therapies, cancer vaccines, tumor-infiltrating lymphocyte (TIL) therapy and immune checkpoint inhibitor (ICI) therapy, recent studies demonstrating that these treatments have the potential to induce lasting tumor remissions [12,13,14,15]. As with all treatments under investigation, numerous limitations persist, primarily due to low response rates and selective efficacy, which are largely attributed to the complex and highly intricate tumor immune microenvironment [16].

With the advancement of research on the tumor microenvironment, various immune cell types have been identified and extensively studied, given that immunocytes constitute the cellular foundation of immunotherapies and are considered essential factors in providing strategies for cancer treatment and for stopping cancer progression. For this reason, decrypting the composition of immune infiltrates in the tumor microenvironment along with their molecular footprint encoded by protein structures on their membranous surface known as Clusters of Differentiation (CD) is necessary for predicting therapeutic response and also for creating novel and highly personalized treatment strategies [17,18,19].

The majority of recently published studies emphasize the role of infiltrating T lymphocytes in different regions of the immune TME due to their high abundance and tumor-suppressive capabilities [20]. Several T lymphocyte subtypes have been described, the most intensely studied being CD8+, CD3+ and CD4+ T lymphocytes. All of these subtypes have been associated in the majority of previous studies with a more favorable prognosis, while FoxP3 expression has been correlated with colorectal cancer progression [21,22,23].

Conversely, comparatively less attention has been directed toward the ‘other side of the immune landscape’—B lymphocytes within the tumor microenvironment—and their potential role in inhibiting carcinogenesis. Until recently, B cells residing in the tumor microenvironment, particularly within tertiary lymphoid structures, a specialized sub-compartment of the TME, have remained insufficiently studied. However, multiple studies published in the past decade have highlighted the potentially significant impact of these cells on cancer immuno-oncology and demonstrated that B lymphocytes in the TME act as antigen-presenting cells, having the capacity to secrete antibodies as well as a several cytokines which directly inhibit the activity of tumor cells. They are also capable of modulating the activity of other cells localized within the tumor microenvironment, such as T lymphocytes, macrophages, and monocytes [24,25,26].

### 1.2. Study Rationale

This systematic review aims to comprehensively identify and evaluate recently published studies concerning the types and functions of tumor-infiltrating B lymphocytes (TIBLs) in colorectal cancer. Few studies have previously investigated the role of TILBs in colorectal carcinogenesis, compared to TILs. Our focus encompasses not only the role of B lymphocytes within the tumor microenvironment but also their presence and function within tertiary lymphoid structures, which constitute a specialized compartment of the TME. This study seeks to clarify the precise interactions between TIBLs and adjacent cellular components within the TME, as well as to assess the prognostic and predictive significance of TIBLs, their correlation with conventional clinicopathological features, and their potential to serve as a foundational reference for the development of future immunologically targeted therapies.

## 2. Materials and Methods

### 2.1. Systematic Search Strategy

We conducted a systematic review adhering to the Preferred Reporting Items for Systematic Reviews and Meta-Analyses (PRISMA) guidelines. The review has been registered on the PROSPERO registration platform, reference number PROSPERO 2025 CRD420251127077. A systematic search of the literature was conducted in PubMed (Medline), Embase and Cochrane Library databases, to identify reports published between January 2000 and July 2025, which addressed Tumor-Infiltrating B Lymphocytes presence in colon and rectal cancer immune microenvironment. In addition, we searched gray literature sources https://scholar.google.com (accessed on 12 June 2025); https://clinicaltrials.gov/ (accessed on 25 June 2025) to identify relevant publications. Initial screening was performed independently by two investigators (G.F and V.F) based on the titles and abstracts of the articles to identify ineligible reports using the Covidence systematic review software, Veritas Health Innovation, Melbourne, Australia. Potentially relevant reports were subjected to a full-text review by two investigators (G.F and V.F.), and the relevance of the reports was confirmed after the data extraction process. Disagreements were resolved by consultation with a third co-author (G.C.D). The search terms were composed as follows: ((“B lymphocytes” OR “B lymphocyte” OR “B-lymphocytes” OR “B cells” OR “B cell” OR “B-cell” OR “B-cells” OR “B cell subtypes” OR “CD73” OR “CD20” OR “CD19” OR “CD21” OR “CD27” OR “CD25” OR “CD38” OR “CD138” OR “CD71” OR “TIBs”) AND (“tumor-associated” OR “tumour-associated” OR “anti-tumor” OR “anti-tumour” OR “tumor-infiltrating” OR “tumor infiltrating” OR “tumour-infiltrating” OR “tumour infiltrating” OR “tumor-infiltrated” OR “tumor infiltration” OR “tumour infiltration” OR “infiltrating” OR “tertiary lymphoid”) AND (“colorectal cancer” OR “rectal cancer” OR “colon cancer” OR “colorectal carcinoma” OR “colon carcinoma” OR “rectal carcinoma” OR “colorectal tumor” OR “colon tumor” OR “rectal tumor” OR “colorectal tumour” OR “colon tumour” OR “rectal tumour”))

### 2.2. Study Selection

We systematically evaluated the retrieved literature for inclusion in this study based on predefined selection criteria. The inclusion criteria were: (i) prospective and retrospective original studies involving patients with colon and rectal cancer; (ii) investigations focusing on tumor-infiltrating B lymphocytes or plasma cells within the immune microenvironment of colon and rectal cancers; (iii) assessment of TIBL or plasma cell subsets; and (iv) availability of full-text articles published in English. In cases of duplicate publications, preference was given to the most recent or highest-quality study. Exclusion criteria encompassed reviews, meta-analyses, editorials, commentaries, authors’ replies, animal and cadaver studies, unpublished meeting abstracts, and case reports; however, reference lists were examined to ensure inclusion of all relevant articles.

### 2.3. Data Extraction and Quality Assessment

The data were independently extracted from each study by two investigators (G.F and V.F) and introduced into an a priori-created datasheet. The extracted information included: study characteristics, number of included cases, TILB detection method, TILB subtype and location, patient demographics, pathological characteristics of the tumors and patient survival outcomes.

### 2.4. PICO Methodology

Our study addressed clearly focused issues, adopting the population, intervention, comparison and outcome (PICO) methodology, as follows:

Population: human patients diagnosed with colon and rectal cancer.

Intervention: we considered all studies that researched TIBL subtypes in the immune tumor microenvironment, as well as tertiary lymphoid structures.

Comparison: Identifying and comparing the role of each B cell subtype and phenotype; assessing and comparing the methodologies used for the identification of TIBLs.

Outcomes: Evaluating the impact of TIBLs on immunotherapy outcomes; assessing the prognostic significance of TIBLs in colorectal cancer through investigating correlations between TILBs and survival parameters (overall survival (OS), disease free survival (DFS), cancer specific survival (CSS)); investigating correlations between distinct B cell subtypes and conventional clinicopathological features of colorectal tumors.

### 2.5. Assessment of Methodological Quality

Two authors (G.F and V.F) independently assessed studies’ quality and the risk of bias using the robvis QUIPS tool version 0.3.0.900, available online at https://mcguinlu.shinyapps.io/robvis/ (accessed on 5 August 2025) [27] The assessed domains included: study participation, study attrition, prognostic factor measurement, outcome measurement, study confounding and statistical analysis and reporting. Disagreements in bias assessment were resolved by consultation with a third co-author (G.C.D).

## 3. Results

### 3.1. Study Selection and Characteristics

**Figure 1:**
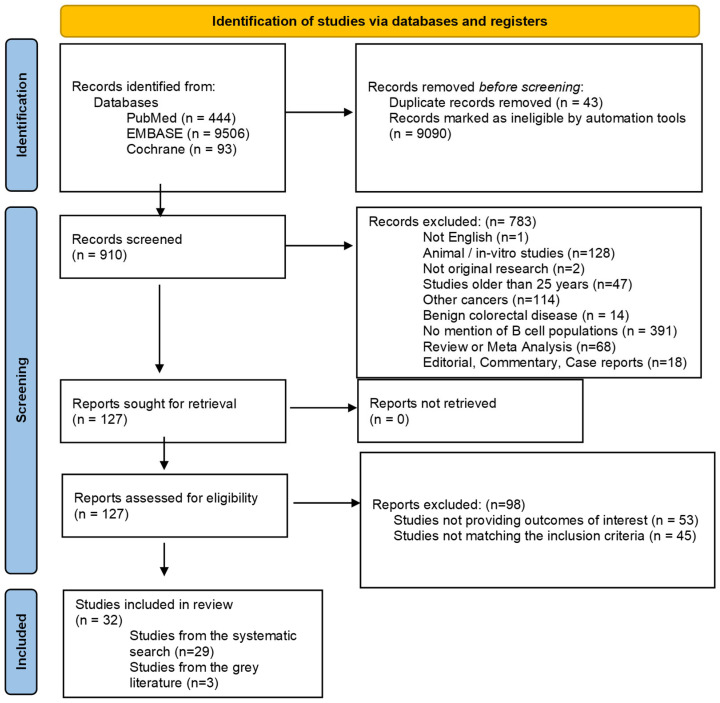
PRISMA flow diagram of the study selection process.

A total of 10,043 records were retrieved from the inquired databases. The literature search identified 910 potentially eligible articles and a total of 32 original papers involving 5766 patients met the inclusion criteria and were included in the current review. The study selection process is outlined in the PRISMA flow diagram (Figure 1).

The characteristics of the included studies, as well as the detection methods employed for the identification of B cell subtypes are presented in Table 1.

### 3.2. Risk of Bias Assessment

**Figure 2:**
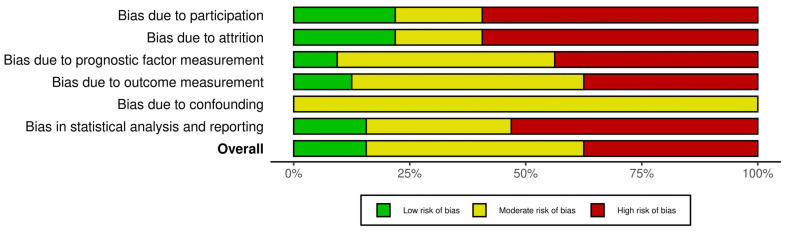
Risk of bias assessment summary.

The proportion of studies judged as having a low risk of bias was as follows: 18.75 % for study participation, 21.87% for study attrition, 9.3% for prognostic factor, 12.5% for outcomes, 0% for confounding and 15.6% for statistical analysis and reporting. The summary analysis of the risk of bias for the included studies is demonstrated in Figure 2. The extended analysis of the risk of bias is presented in Supplementary Figure S1.

### 3.3. Subsets of Tumor-Infiltrating B Lymphocytes

#### 3.3.1. Plasma Cells

Hansen et al. [35] evaluated plasma cells and plasmablasts within the tumor microenvironment of colorectal cancer patients and reported an absence of CD73 expression on plasmablasts. However, a higher surface expression of CD20^+^IgD^−^CD27^+^CD38^+^ was observed, suggesting phenotypic heterogeneity among this subset of B cells, which serve as precursors to plasma cells. In a separate study, Karjalainen et al. [42] identified the expression of B cell markers such as CD20 and CD138, which were found to be transmitted from plasmablasts to mature B cells. These subsets demonstrated prognostic relevance due to their anti-tumor activity within the TME.

#### 3.3.2. Immature Plasma Cell Population Alpha (iMPA)

A study conducted by Xu et al. [29] identified a specific subset of B cells within the colorectal tumor microenvironment, defined as iMPA. This subset, which originates from activated B cells, was found to be significantly associated with the presence of liver metastases in both left- and right-sided colon cancers. The findings suggest a potential role for this B cell population in the metastatic progression of colorectal cancer and highlight its relevance as a possible biomarker for disease dissemination.

#### 3.3.3. Memory B Cells

In a study by Zhong et al. [36], memory B cells were identified as a prominent component within the tumor microenvironment of colorectal cancer. These cells were observed alongside other key immune and stromal populations, including regulatory B cells, T lymphocytes, fibroblasts, and macrophages. The presence of memory B cells within the TME underscores their potential involvement in modulating local immune responses and contributing to the overall immunological landscape of colorectal tumors. Moreover, their coexistence with other immune-regulatory and structural cell types suggests complex cellular interactions that may influence tumor progression, immune evasion, and response to therapy.

#### 3.3.4. Class-Switched Memory B-Cells

Hansen et al. [35] demonstrated that the highest expression of CD73 on tumor-infiltrating B-cells subsets was identified on class-switched memory-B cells, these cells representing a subset of memory B cells that have undergone a process called class-switch recombination this process altering the production of antibodies produced by B cell in TME. Also, they showed that CD73+ B cells expressed significantly lower IgM but higher Ig G compared to CD73− B-cells in the tumor samples and the fact that the staining identified strong enhancement in tumor tissues compared to normal control tissues, the positive CD73 staining being mostly aggregated in the tumor nests.

#### 3.3.5. Naïve B Cells

Naïve B cells were assessed in the studies provided by Hansen et al. [35] and Zhong et al. [36]. Hansen et al. [35] demonstrated that the second highest expression of CD73 on tumor-infiltrating B cells was identified on naïve B-cells which were present in the immune TME of colorectal cancer. Zhong et al. [36] reported that the proportion of naïve B cells was significantly higher in adjacent normal tissue compared to tumor tissue in colorectal cancer patients. This distribution pattern was not observed for other immune cell types, such as CD4^+^ T cells, regulatory T cells (Tregs), and neutrophils, indicating a unique spatial localization of naïve B cells. Furthermore, the study demonstrated that, among all immune cell populations within the tumor microenvironment, only naïve B cells were significantly associated with patient prognosis. According to the current understanding of B-cell development exposed in a comprehensive manner in the study published by Ji et al. [57], naïve B cells differentiated into memory B cells and plasma cells in response to external tumoral antigen stimulation, due to the fact that B cells exhibit distinct genetic differentiation and developmental patterns during colorectal cancer occurrence and development leading to the high heterogeneity of the disease.

Based on the expression of surface markers, the majority of the studies included in the review identified CD20+ B cells [30,31,32,33,34,35,39,40,41,42,43,44,45,46,47,48,49,50,52,53,54,55,56,57]. A small proportion of the included studies identified additional subtypes, such as CD40+, CD27+, CD19+ [28], CD69+ [29], CD23+ [33], CD73+, CD45+ [35,37,39] or CD138+ [41,42,43,49,53]. Yan Mei et al. [28] investigated the proliferative activity and antigen-presenting capacity of CD19+ and CD20+ B cells and found that their proliferation states were higher and more dispersed in tumoral tissue comparing with adjacent and precancerous tissues and that CD40+ and CD27+ B cells had diminished antitumor immunity capacity in CRC.

### 3.4. Identification Methods of Tumor-Infiltrating B Lymphocytes

In order to characterize the landscape of B lymphocytes within the tumor microenvironment of colorectal cancer patients, the majority of studies focused on identifying TIBLs either in isolation or as part of a broader “immune hub” comprising TIBLs in association with T cells (CD4+, CD8+, FOXP3+, CD45RO+), dendritic cells (CD11c+), and macrophages (CD68+). These studies predominantly employed relatively cost-effective detection techniques, such as simple or multiplex immunohistochemistry (IHC) staining on tissue microarrays (TMAs) [31,32,33,34,35,37,39,40,41,42,43,44,45,46,47,48]. In contrast, a smaller number of studies utilized more advanced methodologies, including single-cell RNA sequencing or single-cell ATAC sequencing, which allow for high-resolution profiling of cellular composition and immunological heterogeneity within the tumor immune microenvironment [28]. Other study protocols used multicolor flow-cytometry analysis for the assessment of CD45+ B cells, showing that CD45+CD73+ cell number was significantly increased, whereas the expression of CD73 on CD45+ leucocytes was decreased in CRC tumor tissues in comparison with the paired adjacent normal tissues. CD20+ lymphocytes were assessed in a similar manner, demonstrating a significantly increased density in tumor tissue, an increased cell density of CD20+CD73+ B cells and a decreased expression of CD73 on CD20+ B-cells in tumor tissues [29,35,37,39,45,51].

Zhong et al. [36] and Wu et al. [38] employed the recently developed CIBERSORT algorithm to analyze immune cell populations within colorectal cancer tissues. CIBERSORT enables the deconvolution of high-throughput gene expression data to estimate the relative abundance of specific immune cell types within heterogeneous cell mixtures. This computational tool utilizes defined gene expression signatures to characterize the immune cell composition of complex tissues, offering a robust approach to profiling the tumor immune microenvironment.

Some authors, such as Edin et al. [44], used machine-learning algorithms for tissue segmentation into different tumor compartments (tumor tissue, stromal tissue), cell segmentation and cell phenotyping to identify each of the different immune markers. Qi et al. [50] proposed the use of automatic digital slide scanning of stained tissues, which facilitated the counting of the investigated B cells.

### 3.5. The Prognostic Role of TIBLs on Colorectal Cancer Patients

The majority of studies investigating the prognostic significance of tumor-infiltrating B lymphocytes have reported that elevated TIBL levels are generally associated with improved survival outcomes and a more favorable prognosis in colorectal cancer. However, only three studies [38,57,59] identified a correlation between increased B cell infiltration and poorer prognosis. For instance, Xu et al. [29] demonstrated that, across both metastatic and non-metastatic colorectal cancer patients, a high density of CD19+ B cells was significantly linked to prolonged survival. Moreover, in patients with colorectal cancer liver metastases, elevated CD19+ B cell levels correlated with better clinical outcomes. Similarly, Li et al. [35] reported that reduced CD20+ B cell density within the tumor center was indicative of poorer OS (*p* = 0.028). In a study by Berntsson et al. [41], which examined the tumor microenvironment in relation to tumor sidedness, a high density of CD20+ B cells was associated with improved prognosis in both right- and left-sided colorectal tumors, with increased CD20+ expression significantly correlating with a prolonged five-year OSl.

Edin et al. [44] corroborated previous findings by demonstrating that colorectal tumors with a high infiltration of CD20^+^ B lymphocytes were significantly associated with improved DSS (*p* = 0.001) and overall prognosis. Furthermore, CD20+ B cell infiltration showed a positive correlation with CD8^+^ T lymphocyte presence (*p* < 0.001), suggesting that the prognostic impact results from a cooperative interaction between these lymphocyte subsets. Notably, the prognostic value of CD20+ B cells was evident in non-irradiated colorectal cancer patients but not in those who had undergone radiation treatment for rectal cancer. The study also revealed that patients whose tumors were highly infiltrated by both CD8+ and CD20+ cells exhibited a marginally better prognosis compared to those with high CD8+ but low CD20+ infiltration, indicating a potential supportive role of CD20+ B cells in enhancing CD8+ T cell-mediated anti-tumor immunity. Similar interactions were observed between CD20+ B cells and other immune subsets, including CD66+, CD68+, and FOXP3+ cells.

Qi et al. [50] reported that high densities of CD20+ B cells were associated with increased five-year OS but decreased five-year DFS; however, these findings did not reach statistical significance. According to Nestarenkaite et al. [52] statistically significant patient stratifications were obtained by the Center of Mass indicator for CD20+ B cell densities, for stromal and tumoral aspects CD20+ cell densities, and for intratumoral CD20+ cell density. In the same study, Center of Mass indicator for CD20+ cell density provided 5-year OS rates of 76 and 56%, respectively. Immunogradient-based OS stratifications were similar in the subgroups of MSI and MSS tumors, except for Center of Mass for CD20+ cell density which did not reach statistical significance, this finding indicating a different role of CD20+ cells in MSS tumors. According to the same study, a strong prognostic model was obtained with high CM for CD20+ B cell densities predicting longer patient OS and infiltrative tumor growth pattern independently associated with worse patient survival.

In the study conducted by Berntsson et al. [53], high densities of CD20+, CD138+, and IGKC+ B lymphocytes were found to be significantly correlated with improved five-year OS. Braha et al. [56] reported that a high lymphocytic infiltrate, characterized by CD20+ B cells and CD3+ T cells, was associated with enhanced DFS, although no significant association was observed with overall survival. Furthermore, no statistically significant difference in recurrence rates was detected between lymphocytic infiltrate scores 0–2 and score 3 within the CD20^+^ B cell subgroup. Ji et al. [57] further demonstrated that CD20+ B cells were linked to favorable OS and prognosis across all colorectal cancer samples, with particularly strong associations in right-sided colon cancers. Immunohistochemical staining confirmed a positive correlation between the density of tumor-infiltrating B cells and improved survival outcomes, especially pronounced in patients with right colon tumors.

Hansen et al. [35] reported that increased infiltration of CD73^+^ B cells in colorectal cancer (CRC) tumors was associated with improved overall survival. Similarly, Zhong et al. [36] found that a higher presence of naïve B cells within the tumor microenvironment correlated with better prognosis and enhanced overall survival in CRC patients. Wu et al. [38] conducted an analysis of multiple immune cell populations and demonstrated that both naïve and memory B cells within the TME were significantly associated with colon cancer-related survival outcomes. However, contrasting findings were reported regarding CD73 expression; while Hansen et al. [35] linked higher CD73^+^ B cell infiltration to improved survival, Wu et al. [58] found that elevated CD73 expression was significantly correlated with poorer OS in stage I–III colorectal cancer patients. These results are consistent with those of Lian et al. [59], who also suggested a worse prognosis in CRC patients exhibiting high CD73 expression.

Jiang et al. [46] reported that, in both total-region and normal-region analyses, patients exhibiting larger areas and higher densities of tertiary lymphoid structures (TLSs) experienced significantly improved OS, particularly among non-elderly individuals (*p* = 0.044), males (*p* = 0.002), stage III colorectal cancer cases (*p* < 0.001), patients with low carcinoembryonic antigen levels (*p* = 0.022), and those with both high and low carbohydrate antigen 19-9 levels (*p* = 0.045 and *p* = 0.036, respectively). Among the parameters assessed, total-region TLS density emerged as the most robust prognostic indicator. Zhang et al. [45] demonstrated that TLSs enriched with CD8+ T cells and CD20+ B cells were significantly associated with improved OS. Furthermore, higher intratumoral TLS density correlated with reduced mortality risk, whereas elevated peritumoral TLS density was paradoxically linked to a poorer prognosis. Mori et al. [47] found that the presence of mature TLSs was significantly correlated with prolonged CSS (*p* = 0.0104). In patients with recurrent colorectal cancer, the presence of mature TLSs was also associated with improved post-recurrence OS (*p* = 0.0068). Additionally, high tumoral Ki-67 expression was linked to both poorer OS and DFS. Importantly, the absence of mature TLSs was identified as an independent predictor of reduced post-recurrence OS (*p* = 0.0049). Similarly, Wang et al. [48] reported that low peritumoral TLS density was significantly associated with decreased recurrence-free survival (RFS) and OS. Furthermore, a lower maturation stage of peritumoral TLSs was also significantly correlated with reduced RFS and OS (*p* = 0.010 and *p* = 0.011, respectively). Table 2 illustrates the identified correlations between TILBs and survival outcomes among the included studies.

### 3.6. Relationship Between TIBLs and Clinicopathological Parameters of Colorectal Cancer

In general, high TIBLs were associated with lower TNM stage, lower lympho-vascular invasion and perineural invasion. Xu et al. [29] demonstrated that in stage IV patients, high CD19+ B cells were related to a longer survival time in preoperative chemotherapy treated patients. They also showed that there was a significant decrease in the activated B cell population in primary tumor sites of right colon cancer with liver metastasis compared with right colon cancer without metastasis. Bindea et al. [30] demonstrated that B cell density increased with advancing tumor stage, a trend also observed for neutrophils, mast cells, and macrophages within the tumor immunome. This finding contrasts with the behavior of T cells and diverges from the conclusions of most other studies in the literature.

Shen et al. [31] showed that B cells were highly enriched in the TME compartment in early T tumor stage, so high B cells density was associated with early T stage, thus suggesting a protective role of B cells for limiting extension of the disease. Mao et al. [32] focused on the sidedness of the tumor and demonstrated that in right colon cancer, a high density of B cells is correlated with a lower T stage, a lower positive lymph node rate and a lower positive vascular invasion rate and that patients with a higher number of B cells in tumor microenvironment had a better prognosis. Agoston et al. [33] proved that a higher number of metastatic lymph nodes were associated with significantly lower CD20+. In line with previous studies, Li et al. [34] demonstrated that a reduced presence of CD20+ B lymphocytes at the tumor invasive margin was significantly associated with more advanced TNM stage, increased vascular and perineural invasion, and a higher incidence of distant metastases (*p* < 0.05).

In the study conducted by Hansen et al. [35], patients with metastatic colorectal cancer exhibited a significantly reduced number of tumor-infiltrating CD20+CD73+ B cells, suggesting that a higher density of these B cells may be associated with a lower risk of metastasis. Petrov et al. [37] reported that larger tumors displayed a greater density of B cells, and similarly, poorly differentiated tumors were also characterized by elevated B cell infiltration. In contrast to these findings, Wu et al. [58] observed that high B cell infiltration in colon tumors was associated with poor prognosis, diverging from the conclusions of several previous studies.

Vornhagen et al. [39] reported a decrease in the percentage of IgD+/CD27+/CD38+ naïve B cells with increasing UICC stage. Similarly, Liao et al. [40] found that patients with T1 and T2 stage colorectal cancer had significantly higher densities of CD20+ B cells in the central tumor region (*p* = 0.008). Berntsson et al. [41] also observed that elevated CD20+ B cell infiltration was associated with more favorable tumor characteristics. Specifically, in patients with left-sided colon and rectal cancer, high CD20+ B cell density was significantly associated with lower T stage (*p* = 0.015, *p* < 0.001) and N stage (*p* = 0.046), and in both right- and left-sided tumors, higher CD20+ B cell levels were linked to non-metastatic disease (M0, *p* = 0.002). Moreover, in rectal cancer, high CD20+ B cell infiltration correlated with lower tumor grade. In a study by Zinovkin et al. [43], the rectal tumor stroma of patients with favorable outcomes exhibited significantly higher numbers of TILBs) and IgA plasma cells. IHC analysis revealed dense, rounded infiltrates of CD20+ B cells and clusters of IgA plasma cells near tumor glands. Jiang et al. [46] further demonstrated that low preoperative carcinoembrionary antigen levels were associated with higher TLS and B cell densities, and with absence of lymph node metastasis (*p* = 0.028, *p* = 0.015). Toor et al. [51] supported these findings, reporting that lower levels of tumor-infiltrating B lymphocytes were associated with more advanced disease stages.

Berntsson et al. [53] found that high levels of CD20+ B cells—either isolated or organized in lymphoid aggregates—alongside CD138+ and IGKC^+^+expression, were correlated with lower T stage and negative M stage (both *p* < 0.001). Notably, CD138+ expression was elevated in high-grade tumors and was more frequent in *BRAF* wild-type and microsatellite-stable tumors. Conversely, Wu et al. [58] and Lian et al. [59] reported that high CD73 expression was significantly correlated with adverse clinicopathological features, including elevated preoperative CEA levels, greater tumor depth, poor differentiation, lymph node involvement, and higher AJCC stage.

Trajkowski et al. [54] identified a significant association between high TLS density and lower T stage, particularly at the tumor’s infiltrative front. In contrast, low TLS density was associated with increased lymph node metastasis, greater lymphatic invasion, higher M stage, and poorer tumor differentiation. In a follow-up study, Trajkowski et al. [55] showed that higher densities of tumor-associated lymphocytes, specifically CD20+ B cells, were found in patients with early-stage disease, limited local invasion, and absence of nodal and lymphatic metastasis.

Wang et al. [48] demonstrated that high tumor stroma percentage was significantly associated with lymph node positivity (*p* = 0.0002), increased vascular invasion (*p* = 0.0022), higher TNM stage, and lower organized immune cell aggregate density within non-lymphoid tissues.

### 3.7. Impact of TIBLs on Immunotherapy

Although the primary focus of this review was not the role of tumor-infiltrating B lymphocytes in immunotherapy—given the complexity and breadth of that subject—a limited number of studies have incidentally reported that B cell infiltration may positively influence patient response to immunotherapeutic interventions. Supporting this notion, Mori et al. [47] found that the presence of mature tertiary lymphoid structures was significantly associated with high microsatellite instability (*p* = 0.0081), a condition known to enhance antitumor immune responses. In this context, mature TLSs were defined as organized aggregates of T and B cells containing Ki-67-positive proliferating germinal centers.

Wang et al. [48] observed that elevated peritumoral TLS density was significantly correlated with proficient mismatch repair status and high vascular invasion (*p* = 0.0003 and *p* = 0.0035, respectively), although no significant associations were found with TNM stage, patient sex, age, tumor location, perineural invasion, or histologic grade. Additionally, Lian et al. [59] reported higher CD73 expression levels in mismatch repair-deficient tumors compared to MMR-proficient cases, suggesting that CD73+ B lymphocytes may contribute to reduced responsiveness to immunotherapy in certain colorectal cancer subtypes. Table 3 summarizes the relevant associations between TILBs, pathological characteristics and response to immunotherapy.

### 3.8. Relationship Between B Cells with Other Immune or Non-Immune Cells of the Tumor Microenvironment or Tumoral Markers

Studies reported rich interactions in adjacent and tumor tissues between B cells with T cells, myeloid cells and non-immune cells [28], thus leading to the question of if the function of non-immune cells in shaping immune microenvironment in colorectal cancer is valid. Bindea et al. [30] showed that the expression of markers of Tfh cells in the tumoral immune microenvironment is a cell type known to “help” the generation of B-cell-mediated immune responses, thus underlining the importance of this relation in strengthening the protection against tumor recurrence. They also demonstrated that the immune B cell subsets were found within the tumor at varying cell densities, being of interest to note the predominance of the B cells at the invasive margin compared to the tumor center alongside and in relation with different T cell subsets (CD3, CD45RO, CD8, CD57). Bindea et al. [30] also demonstrated that within the tumor center there was a close correlation between B cells (CD20) and the T cell subset network even though the density of B cells was reduced. Shen et al. [31] demonstrated that B cells alongside with macrophages (TAM2) were necessary in TME for T cells to attack tumor cells.

Li et al. [34] demonstrated that CD20+ B lymphocytes were significantly related to CD68+ macrophages (*p* = 0.004) revealing that CD68+ macrophages could be involved in recruitment of CD20+ B lymphocytes to the surrounding sites and that density of CD 20+ B lymphocytes was higher than CD4+ T lymphocytes, but lower than CD68+macrophages, CD8+ and CD3+ T lymphocytes. Statistically significant intercorrelation between CD20+ B cells and CD3+, CD8+ and FoxP3+ T cell, CD138+ was also demonstrated by Berntsson et al. [41] and Karjalaien et al. [42]. In another study conducted by Berntsson et al. [53] there were significant associations between immune cell-specific expressions of IGKC and CD20 (*p* < 0.001), IGKC and CD 138 (*p* < 0.001) and CD20 and CD138 (*p* < 0.001).

Starting from the premise that regulatory B cells have been proposed to expand regulatory T cells, Vornhagen et al. [39] showed that cytotoxic CD8+ T lymphocytes were both present within and around the tumor while B cells clustered primarily at the invasive margins, the B cells frequently forming follicle-like structures along the tumor border. CD20+ B cells and CD8+ T cells analysis revealed similar colocalization in 40% of the analyzed samples. Ji et al. [57] demonstrated that actions of CD4+ T cells are required for B-cell activation and antibody production process during carcinogenesis at the tumor site. They also showed that B cells primarily interacted with myeloid cells in whole specimens, but more pronounced in tumor tissue than in normal tissue. They also demonstrated that the specific receptor-ligand interactions between B cells and myeloid cells in each region affected the tumor microenvironment exhibiting regional heterogeneity through receptor-ligand interactions between B cells and myeloid cells.

Ji et al. [57] demonstrated that CD20^+^ B cells contribute to modulation of the tumor microenvironment (TME) by facilitating the recruitment and adhesion of other immune cells, particularly CD3^+^ T lymphocytes. Their analysis revealed that CD20^+^ B cells were frequently located in close proximity to CD3^+^ T cells, often forming compact clusters resembling tertiary lymphoid structures. Notably, patients exhibiting high-density co-localized clusters of CD20^+^ B cells and CD3^+^ T cells experienced prolonged survival, highlighting a potential synergistic interaction between B and T lymphocytes—an effect particularly pronounced in right-sided colon cancer. In contrast to these findings, Lian et al. [59] reported that tumor-associated CD73 expression exerted an immunosuppressive effect by inhibiting CD8^+^ T cell activity, thereby promoting carcinogenesis.

In the study conducted by Zhang et al. [45], TLSs were characterized by distinct immune cell compartments, primarily composed of CD3^+^ T cells and CD20^+^ B cells, with B cell follicles representing the predominant structural component. Quantitative analysis of the immune cell populations within TLSs revealed that CD8^+^ T cells were the most abundant (36.16%), followed by CD20^+^ B cells (26.21%). Zhang further described a TLS maturation continuum, ranging from early TLSs—defined as lymphocyte aggregates containing fewer than 50 cells—to primary and secondary TLSs. Primary TLSs exhibited organized morphology, with CD3^+^ T cells encircling CD20^+^ B cell clusters, while secondary TLSs developed germinal centers composed of densely packed T cells surrounding B cell follicles. These distinct maturation stages and the evolving cellular composition of TLSs, particularly the involvement of B cells, were found to play a critical role in shaping the tumor immune microenvironment and influencing colorectal cancer progression. Similarly, Jiang et al. [46] examined the immune composition of mature TLSs and reported a significantly higher density of CD20^+^ B cells compared to non-mature TLSs. Interestingly, mature TLSs did not demonstrate a corresponding increase in CD4^+^ or CD8^+^ T cell density, suggesting that TLS maturation may be primarily driven by B cell infiltration and organization, further underscoring the pivotal role of B cells in modulating local immune responses within the tumor microenvironment.

### 3.9. Spatial Distribution of the TIBLs

Several studies have highlighted the critical role of tumor location in modulating immune responses within the CRC tumor microenvironment. Shen et al. [31] demonstrated that B cells, along with T cells, predominantly localize within the TME compartments, whereas neutrophils and TAM2 macrophages were more frequently distributed in the epithelial compartment. Their analysis further examined the spatial proximity between immune cells and tumor cells by measuring the distance to the nearest malignant cell. Notably, B cells exhibited shorter median distances to tumor cells in early T-stage tumors, suggesting that spatial proximity may serve as an additional mechanism of immune evasion in CRC. Furthermore, their study identified B-cell aggregation in both colon (n = 4) and rectal (n = 3) tumors, with these clusters primarily localized to the tumor-surrounding stromal regions.

Supporting these findings, Mao et al. [32] and Edin et al. [44] reported that CD20^+^ B cell density was significantly higher in right-sided colon tumors compared to left-sided lesions, particularly within the interstitial regions and peripheral tumor tissue. Hansen et al. [35] demonstrated that CD20+ CD73+ B-cell number was significantly elevated in tumors of the rectum. Petrov et al. [37], demonstrated that TIBs were more abundant in colon and sigmoid cancer samples compared with cecal and rectal cancer samples, showing that the median and interquartile range of the TIB fraction were 11.5% and 4–20% in colon cancer, 6% and 3–11% in the sigmoid colon, 2.7% and 0.7–3.7% in cecal cancer and 2.5% and 0.9–3.6% in rectal cancer, respectively.

According to Vornhagen et al. [39] the percentage of CD19+ cells in tumor samples is significantly higher than in peripheral blood of colorectal patients (*p* < 0.05). Also, in the same study the authors concluded that tumor associated B cells contained a higher percentage of CD19+, CD20+ and CD86+ B cells than in peripheral blood or even healthy controls (*p* < 0.005). Also, in the same study CD27+IgD− memory B cells were elevated in colorectal cancer tumor compared to peripheral blood of healthy controls (*p* < 0.005) and IgD+/CD27−/CD38− B cells are decreased in tumor samples of colorectal cancer patients. Also, IHC staining of CD20+ B cells in the tumor microenvironment showed that the predominantly intratumoral distribution of these cells is in lymphoid follicles and TLS. In the same study, the authors demonstrated that CD19+ cells could be detected in all tumor samples and on average made up 8.9% of the CD45+ lymphocytic infiltrate of colorectal cancers compared to 5.1% of the CD45+ peripheral blood. (*p* < 0.005). About one third of the tumor-infiltrating CD19+ cells were CD19+CD20−CD38+ high cells. Also, a well-defined population of cells co-expressing CD19+ and CD20+ could be detected in tumor samples with a non-significant increase in metastatic disease (*p* = 0.28). In the same study the percentage of CD24 high CD38 high (transitional) B cells was lower in tumor samples than in peripheral blood of colorectal cancer patients and transitional B cells were very rare in early-stage CRC (*p* < 0.05). CD24highCD27+ also made up a high percentage of tumor associated B cells in tumor samples.

Zhang et al. [45] conducted an immune infiltration analysis comparing colon cancer tissues with adjacent normal colon tissues and identified significant differences in immune cell composition. The TEM was notably enriched with B cells, alongside CD4^+^ and CD8^+^ T cells, monocytes, and cytotoxic lymphocytes. Wang et al. [48] reported that B cells were distributed throughout the entirety of TLSs, whereas other immune cell types, such as dendritic cells and macrophages, were predominantly localized within the follicular regions. Furthermore, peritumoral TLS density was significantly greater than intratumoral TLS density (*p* < 0.0001). Using single-cell RNA sequencing combined with multicolor immunofluorescence staining, Xia et al. [49] identified five distinct B cell subtypes within the colorectal cancer tumor microenvironment, each characterized by unique marker genes, spatial distribution, and functional properties. They observed a higher proportion of IgG plasma cells in tumor tissues relative to adjacent normal mucosa. Additionally, CD8^+^ T cells were found to facilitate the formation of TLS-associated B cells, with the CCL28–CCR10 chemokine axis playing a crucial role in the migration of IgG plasma cells from the periphery of TLSs into the tumor stroma.

Qi et al. [50] categorized tumor regions into two main subregions: the central tumor and the invasive margin. Each of these was further subdivided into stromal and intratumoral compartments. Consequently, the distribution of TILs was assessed across four distinct compartments: the intratumoral central tumor region, the stromal central tumor region, the intratumoral invasive margin region, and the stromal invasive margin region. In non-dMMR colorectal tumors, CD20^+^ B cells were found to be more concentrated in the stromal central tumor region compared to the stromal invasive margin.

Toor et al. [51] reported that B cells, along with T cells, were abundantly present in both normal colon tissue and the colorectal cancer tumor microenvironment, in contrast to natural killer cells. Although the levels of these immune cells were comparable between tumor and adjacent non-tumor tissues, B cells exhibited a non-significant trend towards reduced abundance within the tumor microenvironment. Given recent evidence that multiple immune checkpoint molecules are expressed on tumor-infiltrating lymphocytes and play critical roles in tumor progression, Toor et al. further examined immune checkpoint expression on B cells. They found that co-inhibitory receptors PD-1, TIM-3, LAG-3, and TIGIT were expressed at relatively low levels on B cells in both tumor and non-tumor tissues, with some indication of increased expression on B cells isolated from tumor tissue. In contrast, the co-stimulatory receptor ICOS was significantly upregulated on B cells within the tumor microenvironment compared to non-tumor tissue.

### 3.10. Neoadjuvant Treatment and Its Influence on B Cells in the Immune Tumor Microenvironment

Hansen et al. [35] demonstrated that neoadjuvant therapy was correlated with reduced CD73+ B-cells in the tumoral microenvironment. Contrary to these findings, Qi et al. [50] showed a slight increase in the infiltration of B cells, along with other cells in the tumor microenvironment from neoadjuvant chemotherapy group compared with non-neoadjuvant chemotherapy group, but there was no significance in the B cell subset (*p* = 0.0624). The results also showed an increase in the infiltration level of B cells in stromal central tumor compartment in post neoadjuvant chemotherapy group. Also, Qi et al. [50] demonstrated results which indicate that neoadjuvant chemotherapy enhanced recruitment of B cells, alongside other immune cells in the stroma of central tumor but not in the invasive margin, which partly justified the small increase in immune infiltration in the overall tumor after chemotherapy. Also, in the study published by Edin et al. [44] preoperative radiotherapy was significantly associated with a reduced infiltration of CD20+ cells.

Emerging evidence suggests that neoadjuvant chemotherapy leads to a marked reduction in B-cell density, accompanied by significant decreases in regulatory T cells, myeloid-derived suppressor cells, natural killer cells, and B lymphocytes [60]. In contrast, an increase in the relative abundance of CD8^+^ T cells has been observed following treatment.

## 4. Discussion

While TNM staging remains a fundamental component of pathological reporting, the density, spatial distribution, and subtype of tumor-infiltrating lymphocytes provide valuable prognostic insights that can deepen our understanding of colorectal cancer behavior and potentially inform more effective, targeted therapeutic strategies [61,62]. Although TNM staging can be readily assessed in resected specimens, evaluating TILs raises concerns regarding time and cost efficiency. Nonetheless, a study addressing the cost-effectiveness of TIL assessment for guiding oncological treatment concluded that incorporating TILs as biomarkers represents a cost-effective strategy [63].

A substantial number of original research and systematic reviews have addressed the prognostic and predictive significance of tumor-infiltrating T lymphocytes in colorectal cancer, as well as in other solid malignancies [64,65]. It is well established from these studies that tumor-infiltrating B lymphocytes are present across various solid tumors, where they may play dual roles—either suppressing or promoting tumor progression. B cells can inhibit tumor growth through mechanisms such as immunoglobulin secretion, stimulation of T cell responses, and direct cytotoxicity against cancer cells. Generally, a high density of B cells correlates with favorable outcomes, although some studies report contradictory findings [66]

B and T cells exhibit both unique and overlapping roles that contribute to a complex immune synergy within the tumor microenvironment of CRC. T cells, particularly CD8+ and CD4+ subsets, mediate direct anti-tumor responses, while B cells support immunity through antigen presentation, antibody production, and TLS formation. However, both regulatory B cells and T cells can exert immunosuppressive effects, highlighting a shared axis of immune regulation [67]. This dual role positions B cells as both enhancers and modulators of T-cell function, with implications for therapeutic strategies targeting adaptive immune coordination in CRC. Recent studies have demonstrated that immunotherapies targeting B-cell maturation antigens show promising clinical efficacy [68].

To address the existing uncertainties and inconsistencies regarding the role of tumor-infiltrating B lymphocytes in colon and rectal cancers, we conducted a systematic review, motivated by the limited information available on these specific cells within the tumor microenvironment. The studies included in our review exclusively involved adenocarcinomas; however, future research should also consider TIBLs in other histological subtypes, such as mucinous carcinoma. Our analysis primarily focused on statistically significant findings reported in the selected studies. Among the 32 included studies, the predominant conclusion is that a high density of TIBLs in colorectal cancer patients is associated with improved prognosis, including enhanced OS, CSS and DFS. Additionally, elevated TIBLs correlated with microsatellite instability-high, deficient mismatch repair status, lower TNM stage, lower tumor grade, and reduced lymphovascular and perineural invasion. These findings align with data on tumor-infiltrating T lymphocytes in colorectal, lung, gastric, esophageal, and breast cancers, supporting the notion of a synergistic antitumor immune response involving both T and B lymphocytes [10].

Due to significant heterogeneity in outcome measures across studies, a meta-analysis was not feasible. Because of several inconsistent outcome measures and the presence of a multitude of confounding factors in the included studies, the results of a meta-analysis could be unreliable and potentially misleading. Further studies should strive towards the application of more uniform protocols for data acquisition.

To facilitate clinical translation, the development of standardized B cell markers and scoring systems is essential. Recent studies underscore the importance of standardized immune evaluation systems in colorectal cancer. The Immunoscore, which quantifies the density and localization of T cells within the TEM, is now validated as a reliable predictor of CRC recurrence risk and patient prognosis [69]. Building on this, emerging efforts are focusing on developing analogous frameworks for B cells, which may enhance the precision of immune-based risk stratification in CRC.

Furthermore, prospective, high-quality studies are warranted, with an emphasis on elucidating the role of TIBLs in emerging immunotherapies, including cancer vaccines, chimeric antigen receptor cell therapies, and tumor-infiltrating lymphocyte therapies, which are currently under extensive investigation [14]. Future research should aim towards identifying therapeutic strategies which enhance the beneficial functions of B cells in the TME while simultaneously blocking the immunosuppressive roles of regulatory B cells, particularly in combination with existing immunotherapies. The presence of certain B cell subsets could be useful as specific biomarkers for successful immune responses to anti-cancer vaccines, contributing to the development of novel targeted therapeutic strategies. In addition, future efforts should be directed towards validating standardized protocols for Immunoscores based on B cells, improving the characterization of B cell functions and stratifying response according to MSI status.

TIBLs in CRC exhibit altered clonotypes, phenotypes, and immunoglobulin subclasses compared to those in adjacent normal tissue. Notably, these tumor-associated immunoglobulin signatures are also detectable in patient plasma, indicating a systemic B cell response. A recently proposed plasma-based diagnostic model demonstrated improved sensitivity when compared to traditional biomarkers (CEA and CA19-9), highlighting the potential of circulating immunoglobulin signatures as a non-invasive tool for CRC detection [70].

To the best of our knowledge, this represents the first systematic review specifically examining the role of tumor-infiltrating B lymphocytes in colon and rectal cancer patients. However, several limitations should be noted. Firstly, not all studies assessed the same clinical outcomes. Secondly, some studies did not specify whether patients received neoadjuvant chemotherapy. Thirdly, the inclusion of full-text articles published in English may introduce a language bias to the present study. Additionally, the included studies combined colon and rectal cancer cases, which may obscure site-specific differences. Future studies should aim to analyze larger and more homogenous cohorts of patients. Lastly, the majority of studies lacked standardized methods for reporting and quantifying TIBLs, underscoring the need for uniformity in future investigations.

## 5. Conclusions

In summary, TIBLs in colorectal cancer are attracting great interest among researchers in the field of immuno-oncology. This systematic review identified a favorable prognostic role of high density TIBLs among patients with CRC, providing a rationale for further accurate predictive and prognostic analysis. The spatial distribution variations in the immune TME as well as changes in the density of TIBLs also impact prognosis, highlighting the complexity of the tumor microenvironment cellular multiverse. Standardized reporting and quantification methods for TILBs should be considered in future studies in order to obtain more robust conclusions. Integrating spatial metrics and functional characterization of B cells into future research may allow for the identification of novel tissue-specific biomarkers, in order to better guide treatment decisions.

## Figures and Tables

**Table 1:** Characteristics of the included studies, patient populations and detecting methodology.

Study	Year of Publication	Sample Size	Tumor Location	Neoadjuvant Treatment	Detecting Method
Yan Mei et al. [28]	2021	12	Colon and rectum	No	Single-cell RNA-sequencing, single-cell ATAC-sequence
Xu et al. [29]	2022	401	Colon and rectum	Mixed population (Yes and no)	TMA, single-cell RNA-sequencing, IHC, multiplexed IHC, IF staining, flow cytometry
Bindea et al. [30]	2013	105	Colon and rectum	n.r.	Real-time PCR, TMA
Shen et al. [31]	2024	190	Colon and rectum	No	Multiplex IHC, TMA
Mao et al. [32]	2023	128	Colon	No	HE, IHC
Agoston et al. [33]	2022	137	Colon and rectum	n.r.	IHC, TMA
Li et al. [34]	2017	419	Colon and rectum	n.r.	IHC
Hansen et al. [35]	2022	62	Colon and rectum	Yes	HE, IHC, multicolor flow cytometry
Zhong et al. [36]	2022	232	Colon and rectum	n.r.	CIBERSORT, multiplex immunofluorescence staining
Petrov et al. [37]	2024	31	Colon and rectum	No	IHC, flow cytofluorometry
Wu et al. [38]	2020	369	Colon	n.r.	CIBERSORT
Vornhagen et al. [39]	2014	38	Colon and rectum	n.r.	Flow cytometry, IHC
Liao et al. [40]	2013	67	Colon and rectum	n.r.	IHC, TMA
Berntsson et al. [41]	2017	557	Colon and rectum	n.r.	TMA, IHC, HE
Karjalainen et al. [42]	2023	221	Colon and rectum	No	IHC, TMA
Zinovkin et al. [43]	2021	155	Rectum	Yes	IHC
Edin et al. [44]	2019	275	Colon and rectum	Yes	Multiplex IHC, TMA, multispectral imaging
Zhang et al. [45]	2025	374	Colon	No	Multispectral IHC, flow cytometry
Jiang et al. [46]	2025	114	Rectum	Yes	Multiplex IHC, Strataquest
Mori et al. [47]	2024	78	Colon and rectum	No	IHC
Wang et al. [48]	2022	114	Colon and rectum	No	HE, IHC
Xia et al. [49]	2023	14	Colon and rectum	n.r.	Single-cell RNA-sequencing, multicolor immunofluorescence staining flow cytometry
Qi et al. [50]	2021	77	Colon and rectum	Yes	Multiplex IHC staining
Toor et al. [51]	2021	50	Colon and rectum	No	Flow cytometry
Nestarenkaite et al. [52]	2020	87	Colon and rectum	n.r.	IHC
Berntsson et al. [53]	2016	626	Colon and rectum	n.r.	IHC, TMA
Trajkovski et al. [54]	2018	103	Colon and rectum	n.r.	IHC
Trajkovski et al. [55]	2018	103	Colon and rectum	n.r.	IHC
Braha et al. [56]	2015	195	Colon and rectum	n.r.	IHC
Ji et al. [57]	2024	209	Colon and rectum	n.r.	Single-cell RNA-sequencing, IHC, immunofluorescence staining
Wu et al. [58]	2012	223	Colon and rectum	n.r.	IHC, TMA, Western blot
Lian et al. [59]	2024	n.r.	Colon and rectum	n.r.	IHC, immunofluorescence staining, Western blot, flow cytometry

n.r.—not reported; IHC—immunohistochemistry; RNA—ribonucleic acid; ATAC—assay for transposase-accessible chromatin; TMA—tissue microarrays; IF—Immunofluorescence; HE—Hematoxylin-eosin.

**Table 2:** Characteristics of TILB location, subsets and correlations with survival parameters.

Study	B Cell Location	Investigated B Cell Subsets	Correlation with Other Immune Subsets	Correlation with OS	Correlation with DFS	Correlation with CSS	Prognostic Value of TIBLs
**Yan Mei et al.** [28]	adjacent to tumor, precancerous and tumoral tissue	B cells- CD 19+, CD20+, CD40+, CD27+, KLRB1+, CCL5+ and plasma cells- MZB1+, DUSP1+, CCL3+	T cells, myeloid cells and non-immune cells	n.r.	n.r.	n.r.	n.r.
**Xu et al.** [29]	n.r.	CD19+, CD69+	n.r.	High CD19, CD69 = high OS	High CD19, CD69 = high DFS	High CD19, CD69 = high CSS	High level of CD19, CD69 = good prognosis in patients with liver metastasis
**Bindea et al.** [30]	center of the tumor, invasive margin	CD20+	Tfh, memory T cells	High CD20 = high OS	High CD20 = high DFS	High CD20 = high CSS	High level of CD20 = good prognosis
**Shen et al.** [31]	TME compartment, epithelial compartment	CD20+	TAM2, T cells	High CD20 = high OS	n.r.	n.r.	High level of CD20 = good prognosis
**Mao et al.** [32]	center of the tumor, invasive margin	CD20+	CD4 T, CD 8 T, CD45RO T, CD21 follicular dendritic, CD11 dendritic cell, CD15 granulocytes, CD68 macrophages, FOXP3 Treg cells, NCR1+	High CD20 = high OS	n.r.	n.r.	High level of CD20 = good prognosis
**Agoston et al.** [33]	main tumor mass, tumor normal interface, deepest infiltrative area	CD20+, CD23+	n.r.	n.r.	n.r.	n.r.	n.r.
**Li et al.** [34]	tumor center, tumor invasive margin	CD20+	CD68 +macrophages	Low CD20 = low OS	n.r.	n.r.	High level of CD20 = good prognosis
**Hansen et al.** [35]	tumor center, tumor invasive margin	CD73+(class-switched memory B cell, naive B-cells), CD45+, CD20+	CD 73	High CD 73 = high OS	High CD73 = high DFS	High CD73 = high CSS	High level of CD73 = good prognosis
**Zhong et al.** [36]	tumor center, tumor invasive margin	Memory B cells, naïve B cells	n.r.	High naïve B cells = high OS	n.r.	n.r.	High naïve B cell count = good prognosis
**Petrov et al.** [37]	tumor center, tumor invasive margin	CD19, CD45	n.r.	n.r.	n.r.	n.r.	n.r.
**Wu et al.** [38]	n.r.	Memory B cells, naïve B cells	n.r.	High B cell count = low OS	High B cell count = low DFS	High B cell count = low CSS	High B cell count = poor prognosis
**Vornhagen et al.** [39]	tumor center, tumor invasive margin, peripheral blood, liver metastasis	CD19+, CD45+, CD20+, CD38+, CD27+ naïve and memory B cell	Ki67, T cells	n.r.	n.r.	n.r.	n.r.
**Liao et al.** [40]	tumor center, tumor invasive margin	CD20+	n.r.	n.r.	n.r.	n.r.	n.r.
**Berntsson et al.** [41]	intratumoral, tumor-adjacent, distant stroma	CD20+, CD138+, IGKC+	CD3+, CD8+ T cells, Foxp3, CD138+	High B cell count = high OS	n.r.	n.r.	High B cell count = good prognosis
**Karjalainen et al.** [42]	tumor center, tumor invasive margin	CD20+, CD138+	CD3+, CD8+ T cells	ns	n.r.	High B cell count = high CSS	High B cell count = good prognosis
**Zinovkin et al.** [43]	rectal cancer stroma	CD20+, CD 138+ (Ig A+ infiltrating plasma cells)	CD3+ T cells	High B cell count = high OS	High B cell count = high DFS	High B cell count = high CSS	High B cell count = good prognosis
**Edin et al.** [44]	tumor center, tumor invasive margin	CD20+	CD8+ T cells	n.r.	n.r.	High B cell count = high CSS	High B cell count = good prognosis
**Zhang et al.** [45]	intratumoral, peritumoral	CD20+	CD4+, CD8+ T cells	High B cell count = high OS	High B cell count = high DFS	High B cell count = high CSS	High B cell count = good prognosis
**Jiang et al.** [46]	tumor, normal tissue	CD20+	CD4+, CD8+, CD 21 +, CD23+	High B cell count = high OS	n.r.	n.r.	High B cell count = good prognosis
**Mori et al.** [47]	tumor center, tumor invasive margin	CD20+	CD3+, FoxP3, CD8+ T cells and macrophages, Ki-67	ns	ns	Mature TLS = high CSS	High B cell count = good prognosis
**Wang et al.** [48]	peritumoral, intratumoral	CD20+	CD4+, CD8+, CD45RO+, CD11c+, CD68+	Low B cell count = low OS	Low B cell count = low DFS	n.r.	High B cell count = poor prognosis
**Xia et al.** [49]	tumor center, tumor invasive margin	CD20+, CD138+, CD27+, CD38	CD8+ T cells	n.r.	n.r.	n.r.	n.r.
**Qi et al.** [50]	tumor center, tumor invasive margin	CD20+	CD4+, CD8+ T cells, CD68+ macrophages, CD66b+ granulocytes	ns	ns	n.r.	Not statistically significant
**Toor et al.** [51]	tumor tissue and corresponding non-tumoral tissue	CD19+	CD4+, CD8+	ns	ns	n.r.	Not statistically significant
**Nestarenkaite et al.** [52]	intratumoral tissue, inside the interface zone (tumor, tumor edge, stroma)	CD20+	CD8+ T cells, CD68+ macrophages	High B cell count = high OS	n.r.	n.r.	High B cell count = good prognosis
**Berntsson et al.** [53]	intratumoral, within the adjacent stroma and within the distant stroma	CD20+, CD138+, IGKC+	CD138+, IGKC	High B cell count = high OS	n.r.	n.r.	High B cell count = good prognosis
**Trajkovski et al.** [54]	tumor center, tumor invasive margin	CD20+, CD21+	CD4+, CD8 + T cells	n.r.	n.r.	n.r.	n.r.
**Trajkovski et al.** [55]	tumor center, tumor invasive margin	CD20+	CD8+, CD4+ T cells	n.r.	n.r.	n.r.	n.r.
**Braha et al.** [56]	tumor center, tumor invasive margin	CD20+	CD3+ T cells	ns	High B cell count = high DFS	n.r.	High B cell count = good prognosis
**Ji et al.** [57]	tumor center, tumor invasive margin	CD20+	Myeloid cells, CD3+ T cells	High B cell count = high OS	High B cell count = high DFS	High B cell count = high CSS	High B cell count = good prognosis
**Wu et al.** [58]	tumor center, tumor invasive margin	CD73+	n.r.	High B cell count = low OS	High B cell count = low DFS	High B cell count = low CSS	High B cell count = poor prognosis
**Lian et al.** [59]	tumor center, tumor invasive margin	CD73+	CD8+ T cells	High B cell count = low OS	n.r.	n.r.	High B cell count = poor prognosis

n.r—not reported; ns—not statistically significant; OS—overall survival; DFS—disease free survival; CSS—cancer specific survival; IGKC—immunoglobulin K constant.

**Table 3:** Correlations between pathological characteristics and TILB expression.

Study	Correlation with T Stage	Correlation with N Stage	Correlation with M Stage	Correlation with V and Pn Stage	Prediction to Immunotherapy Response	Correlation with MSI Status
**Yan Mei et al.** [28]	n.r.	n.r.	n.r.	n.r.	B cell enriched tumors = good response to immunotherapy	n.r.
**Xu et al.** [29]	High CD19, CD69 = low T stage	n.r.	Low B cell count in primary tumor = liver metastasis	n.r.	n.r.	n.r.
**Bindea et al.** [30]	High B cell count = high T stage	n.r.	n.r.	n.r.	n.r.	n.r.
**Shen et al.** [31]	High B cell count = low T stage	n.r.	n.r.	n.r.	n.r.	n.r.
**Mao et al.** [32]	High B cell count = low T stage	high B cell lower positive lymph node rate	n.r.	High B cell count = low V stage	n.r.	n.r.
**Agoston et al.** [33]	High CD45+ = high T stage	low B cell count high number of metastatic lymph node	High B cell count in liver and lymph node metastatic tumor	n.r.	n.r.	n.r.
**Li et al.** [34]	Low CD20+ = high T stage	lob CD20+ B cell count high number of metastatic lymph node	Low CD20+ = distant metastasis	Low CD20+ B cell count = high V and Pn stage	n.r.	n.r.
**Hansen et al.** [35]	Low CD73 = high T stage	No difference in LN+ and LH negative regarding the density of CD20+CD73+	Low CD73 = liver metastasis	n.r.	n.r.	n.r.
**Zhong et al.** [36]	ns	ns	ns	ns	n.r.	n.r.
**Petrov et al.** [37]	ns	ns	ns	n.r.	n.r.	n.r.
**Wu et al.** [38]	High B cell count = high T stage	High B cell high N stage	ns	n.r.	n.r.	n.r.
**Vornhagen et al.** [39]	Low naïve B cell count = high T stage	low naïve B cell = high N stage	Low naïve B cell count = high M stage	n.r.	n.r.	n.r.
**Liao et al.** [40]	High B cell count = low T stage	n.r.	n.r.	n.r.	n.r.	n.r.
**Berntsson et al.** [41]	High B cell count = low T stage	High B cell low N stage	High B cell count in nonmetastatic patients	n.r.	n.r.	n.r.
**Karjalainen et al.** [42]	ns	ns	ns	ns	n.r.	ns
**Zinovkin et al.** [43]	ns	ns	ns	n.r.	B cell enriched tumors = good response to immunotherapy	n.r.
**Edin et al.** [44]	High B cell count = low T stage	High B cells low stage	High B cell count = low M stage	n.r.	n.r.	n.r.
**Zhang et al.** [45]	High B cell count = low T stage	n.r.	n.r.	n.r.	n.r.	n.r.
**Jiang et al.** [46]	High B cell count = low T stage	High B cells low N stage	High B cell count in nonmetastatic patients	High B cell count = low V stage	n.r.	n.r.
**Mori et al.** [47]	ns	ns	ns	n.r.	n.r.	Mature TLS = positive MSI status
**Wang et al.** [48]	ns	ns	ns	High TLS count = low V stage	n.r.	High TLS = positive MSI status
**Xia et al.** [49]	n.r.	n.r.	n.r.	n.r.	B cell enriched tumors = good response to immunotherapy	High B cell counts = positive MSI status
**Qi et al.** [50]	ns	ns	ns	ns	n.r.	n.r.
**Toor et al.** [51]	Low B cell count = high T stage	n.r.	n.r.	ns	B cell enriched tumors = good response to immunotherapy	ns
**Nestarenkaite et al.** [52]	ns	n.r.	ns	ns	n.r.	High B cell counts = positive MSI status
**Berntsson et al.** [53]	High B cell count = low T stage	ns	High B cell count = low M stage	ns	n.r.	High B cell counts = negative MSI status
**Trajkovski et al.** [54]	High B cell count = low T stage	low TLS = high N stage	Low TLS count = high M stage	Low TLS count = high V stage	n.r.	n.r.
**Trajkovski et al.** [55]	Low B cell count = high T stage	low TAL = high N stage	Low B cell count = distant metastasis	Low TAL count = high V stage	n.r.	n.r.
**Braha et al.** [56]	Low B cell count = high T stage	Not statistically significant	Low B cell count = distant metastasis	Not statistically significant	n.r.	n.r.
**Ji et al.** [57]	n.r.	n.r.	Low B cell count = liver metastasis	n.r.	n.r.	n.r.
**Wu et al.** [58]	High B cell count = high T stage	High B cell high nodal status	High B cell count = high M stage	n.r.	n.r.	n.r.
**Lian et al.** [59]	High B cell count = high T stage	n.r.	High B cell count = liver metastasis	n.r.	Low B cell count = good response to immunotherapy	High B cell count = positive MMRd status

n.r.—not reported; ns—not statistically significant; TLS—tertiary lymphoid structure; TAL = tumor associated lymphocytes; MSI—microsatellite instability; MMRd—mismatch repair deficient.

## Data Availability

Data concerning this study is available upon reasonable request from the corresponding author.

## Supplementary Materials

The following supporting information can be downloaded at: https://www.mdpi.com/article/10.3390/cancers17182996/s1, Figure S1: Risk of bias assessment for each of the included studies.

## Abbreviations

The following abbreviations are used in this manuscript:

TIL	Tumor-infiltrating lymphocyte
TIBL	Tumor-infiltrating B lymphocyte
TME	Tumor microenvironment
CAR-T	Chimeric antigen receptor T
CD	Cluster of differentiation
n.r.	Not reported
Ns	Not statistically significant
IHC	Immunohistochemistry
RNA	Ribonucleic acid
ATAC	Assay for Transposase-Accessible Chromatin
TMA	Tissue microarrays
IF	Immunofluorescence
HE	Hematoxylin-eosin
CRC	Colorectal cancer
IGKC	Immunoglobulin Kappa constant
TAL	Tumor associated lymphocyte
TLS	Tertiary lymphoid structure
MSI	Microsatellite instability
OS	Overall survival
DFS	Disease free survival
CSS	Cancer-specific survival
