# Supplementary material for: The Role of Tumor-Infiltrating B Lymphocytes in Colorectal Cancer Patients: A Systematic Review of Immune Landscape Evolution
Source: Cancers (Basel). 2025 Sep 13;17(18):2996. doi: 10.3390/cancers17182996 (PMC12468775; doi:10.3390/cancers17182996)
Supplement: Supplementary file 1 [file cancers-17-02996-s001.zip › cancers-3847424-supplementary.pdf]

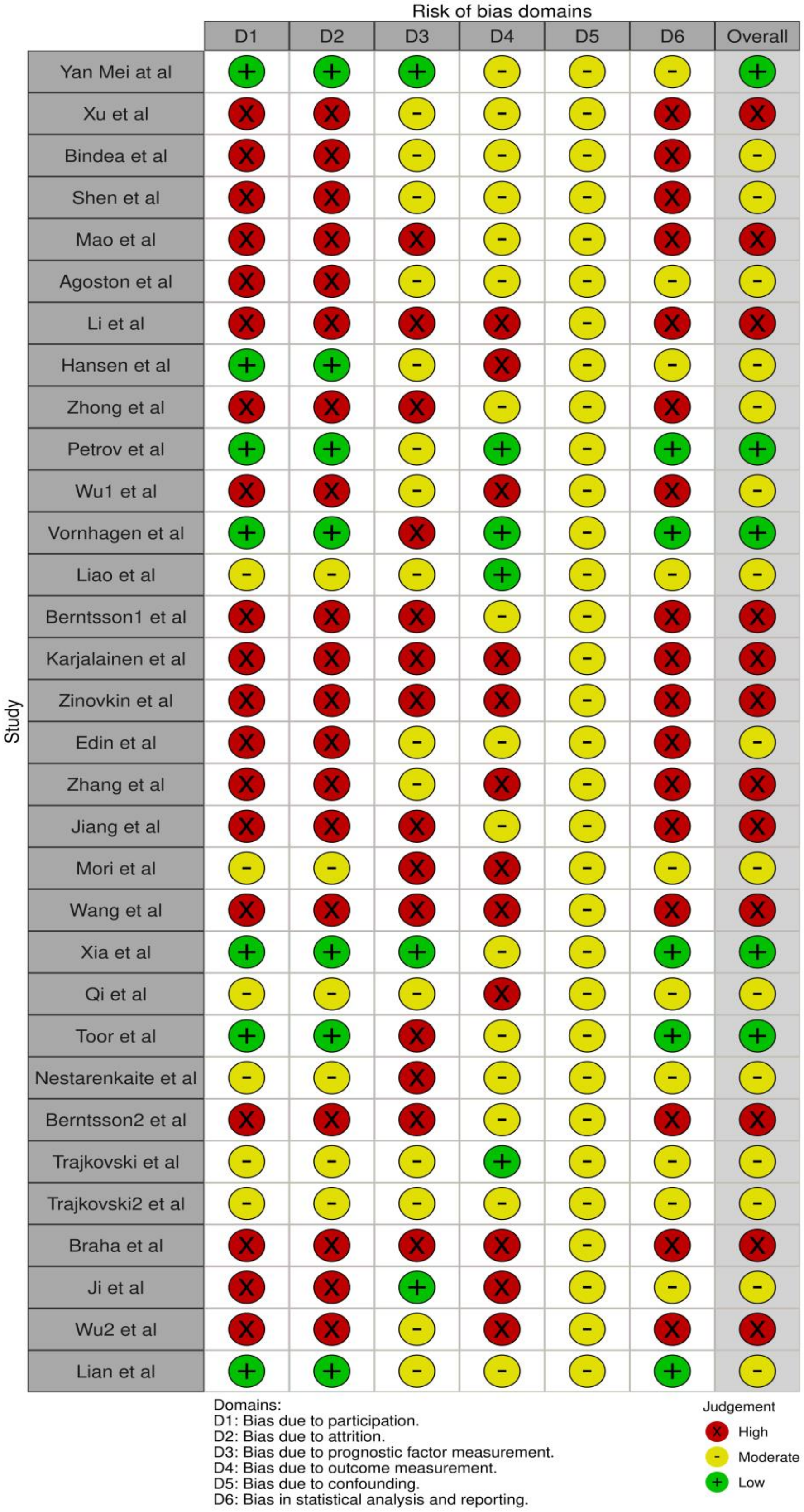

Supplementary Figure S1. Risk of bias assessment for each of the included studies.
